# Pattern and Forensic Significance of Defense Injuries in Homicide Cases: A Cross-Sectional Study

**DOI:** 10.7759/cureus.79959

**Published:** 2025-03-03

**Authors:** Nani Gopal Das, Nirmalendu Das, Abhik Sil

**Affiliations:** 1 Forensic Medicine and Toxicology, Tripura Medical College and Dr. Bhimrao Ramji Ambedkar Memorial Teaching Hospital, Agartala, IND; 2 Health and Family Welfare, Kendriyo Sanshodhanagar, Tripura, IND; 3 General Surgery, Tripura Medical College and Dr. Bhimrao Ramji Ambedkar Memorial Teaching Hospital, Agartala, IND

**Keywords:** crime reconstruction, defense injuries, forensic pathology, homicide investigation, weapon analysis

## Abstract

Introduction

Defense injuries play a crucial role in forensic investigations by providing insights into the nature of an assault, the type of weapon used, and the manner of death. This study aims to analyze the prevalence, patterns, and forensic significance of defense injuries in homicide cases.

Methods

A retrospective observational study was conducted on 60 homicide cases with documented defense injuries. Data was collected from autopsy reports, crime scene records, and inquest papers over a five-year period at a tertiary care hospital and compared. The injuries were categorized based on type, anatomical distribution, and the weapon involved. Statistical analysis was performed to assess the relationship between defense injuries and weapon type.

Results

Defense injuries were most commonly observed on the hands 48 (80%), forearms 39 (65%), and fingers 24 (40%). Incised wounds were predominant 36 (60%) in knife attacks, whereas contusions and fractures were frequently seen 33(55%) in blunt force trauma cases. Gunshot victims showed fewer defense injuries 12 (20%), often in the form of abrasions or contusions. The analysis indicated a significant correlation between the type of weapon and the nature of defense wounds (p<0.05).

Conclusion

The study highlights the forensic importance of defense injuries in homicide cases, aiding in crime scene reconstruction and medico-legal investigations. Proper documentation of these injuries can strengthen legal proceedings and ensure justice.

## Introduction

Defense injuries, also known as defensive wounds, occur when a victim attempts to shield themselves from an attacker. It results from the immediate and instinctive reaction of the victim to ward off an attack in order to ensure survival [1]. These injuries are crucial in forensic medicine practice in homicide investigations throughout the world, providing insights into the mode of assault, the weapon used, and the comparative power of the assailant and victim [2].

Understanding the pattern and distribution of these injuries helps differentiate homicide from suicide and accidental injuries, thereby influencing legal interpretations. Forensic experts consider defense injuries an essential element in reconstructing the sequence of events leading to death. The anatomical location, type, and severity of such injuries play a crucial role in understanding the circumstances of the crime [3].

As of data till December 2022, the National Crime Records Bureau (NCRB) displayed that a total of 28,522 murder cases were registered across India, averaging approximately 78 incidents per day. Uttar Pradesh reported the highest number of murder cases at 3,491, while Sikkim had the fewest with just nine cases [4].

In many homicide cases, the presence of defense injuries indicates victim resistance, while their absence might suggest restraint, incapacitation, or a surprise attack. Studies have shown that in 79% of cases of homicide, upper limbs, particularly the hands and forearms, are the most common sites of defense wounds. Analyzing these injuries in detail enhances the ability of forensic pathologists to infer the victim’s actions during the assault [5].

This study investigates the patterns and forensic significance of defense injuries in homicide cases to enhance the understanding of forensic experts and law enforcement agencies.

## Materials and methods

Study design and setting

This study was a retrospective cross-sectional study with a descriptive observational design. It was conducted in the Department of Forensic Medicine and Toxicology, Tripura Medical College and Dr. Dr. Bhimrao Ramji Ambedkar Memorial Teaching Hospital, Hapania, Agartala, West Tripura, India. The study period spanned five years, from January 2019 to December 2024.

Study materials and data sources 

Data were obtained from medico-legal autopsy reports in the Forensic Medicine and Toxicology department's records, with prior administrative approval, crime scene records from the investigating police officer, and medico-legal documents at the casualty.

Sample size and selection criteria

A total of 60 confirmed homicide cases were included in the study. The required sample size was calculated using the following formula: n=Z^2^×p×(1−p)/d^2^,where Z=1.96 (Z-score for a 95% CI), p=0.5 (expected prevalence based on previous studies), and d=0.13 (acceptable margin of error). The calculated sample size is approximately 57 cases. Since sample sizes are usually rounded up for feasibility, a sample size of 60 cases is justified. This ensures adequate statistical power while maintaining practical constraints (limited cases) in forensic medicine studies. Cases were selected based on specific inclusion and exclusion criteria to ensure forensic relevance. Only confirmed homicide cases with documented defense injuries and detailed autopsy reports were included. Cases with complete medico-legal records were prioritized to ensure data accuracy and reliability. Exclusions included unidentified bodies with incomplete records, decomposed bodies where injury assessment was compromised, and cases where the manner of death was undetermined.

Sampling method

A purposive sampling method was employed. Given the relatively lower number of homicide cases, this method was used to specifically include cases that met the study’s forensic requirements. Only those cases with clear documentation of injury patterns were selected to ensure a focused and reliable analysis.

Study tools and data collection procedure

Data were collected using a semi-structured questionnaire to document key forensic variables. Victim demographics, including age, sex, and occupation, were recorded. The cause and manner of death were categorized by homicide method, such as stabbing, blunt force trauma, firearm injury, or strangulation. Defense injuries were classified based on type (incised wounds, lacerations, contusions, and fractures) and anatomical distribution (hands, forearms, fingers, arms, legs, and face). Weapon characteristics (knife, blunt object, firearm, ligature) and injury severity (single vs. multiple wounds) were also analyzed. Data were extracted from autopsy reports, crime scene records, and medico-legal documents, ensuring accuracy. Only comprehensive cases were included, providing a detailed forensic analysis of injury patterns in homicide cases.

Statistical analysis

The available data were entered into MS Excel 2007 (Microsoft Corp., Redmond, WA, US), and then in the software Statistical Package for Social Sciences (SPSS), version 21 (SPSS, Inc., Chicago, IL). The qualitative data were expressed in terms of percentage, and quantitative data were expressed in terms of frequency, mean, and standard deviations. We conducted a chi-square test to determine the association of victims’ demographic details with independent variables like sex, age, religion, educational status, occupation, marital status, and place of residence and to check a statistically significant correlation between weapon type and injury patterns. A p-value of less than 0.05 at a 95% CI is considered statistically significant, leading to the rejection of the null hypothesis in favor of the alternative hypothesis.

Ethical considerations

This study was conducted following ethical principles and guidelines for research involving human subjects. Ethical approval was obtained from the Ethics Committee of Tripura Medical College and Dr. Bhimrao Ramji Ambedkar Memorial Teaching Hospital (12/24), and the study adhered to the principles outlined in the Declaration of Helsinki (2013). The Institutional Ethics Committee consists of a legal representative including all other members. Since this was a retrospective forensic study, informed consent from participants was not required, as no direct interaction with individuals occurred, and only de-identified autopsy records were analyzed. Strict confidentiality measures were maintained, ensuring that all personal identifiers were removed from forensic reports, and the data were used solely for research purposes.

## Results

A significant proportion (42 (70%)) of the cases occurred in individuals aged 20-50 years, suggesting that this age group is at higher risk for such incidents. Male predominance (42 (70%)) was observed among the victims, with females comprising 18 (30%) of the cases. A higher incidence of defense wound cases was observed among Hindus (35, 58%), followed by Muslims (21, 35%), based on the data analysis in the study. A total of 31 (52%) of the victims had no formal education, suggesting a possible correlation between literacy levels and vulnerability. Individuals from unskilled occupational backgrounds comprised 40 (67%) of the victims, indicating higher vulnerability. Married individuals constituted 35 (58%) of the victims, suggesting possible socio-demographic influences on vulnerability. Rural residents accounted for 41 (68%) of the victims, suggesting higher vulnerability in these areas, as shown in Table 1.

**Table 1:** Demographic data analysis (n=60)

Categorical variables	Frequency	Percentage	p-value
Age group (in years)			0.08
0-20	06	20	
20-50	42	70	
>50	12	10	
Gender			0.01
Male	42	70	
Female	18	30	
Religion			0.01
Hindu	35	58	
Muslim	21	35	
Christian	3	5	
Buddhist	1	2	
Educational status			0.02
Illiterate	31	52	
Primary	16	27	
10th	09	15	
12th	03	05	
Graduation	01	01	
Occupation			0.01
Unskilled	40	67	
Skilled	14	23	
Semi-skilled	4	06	
Semi-professional	2	04	
Marital status			0.03
Married	35	58	
Unmarried	19	31	
Divorced	4	7	
Widower	2	4	
Place of residence			0.04
Rural	41	68	
Urban	19	32	

**Figure 1:**
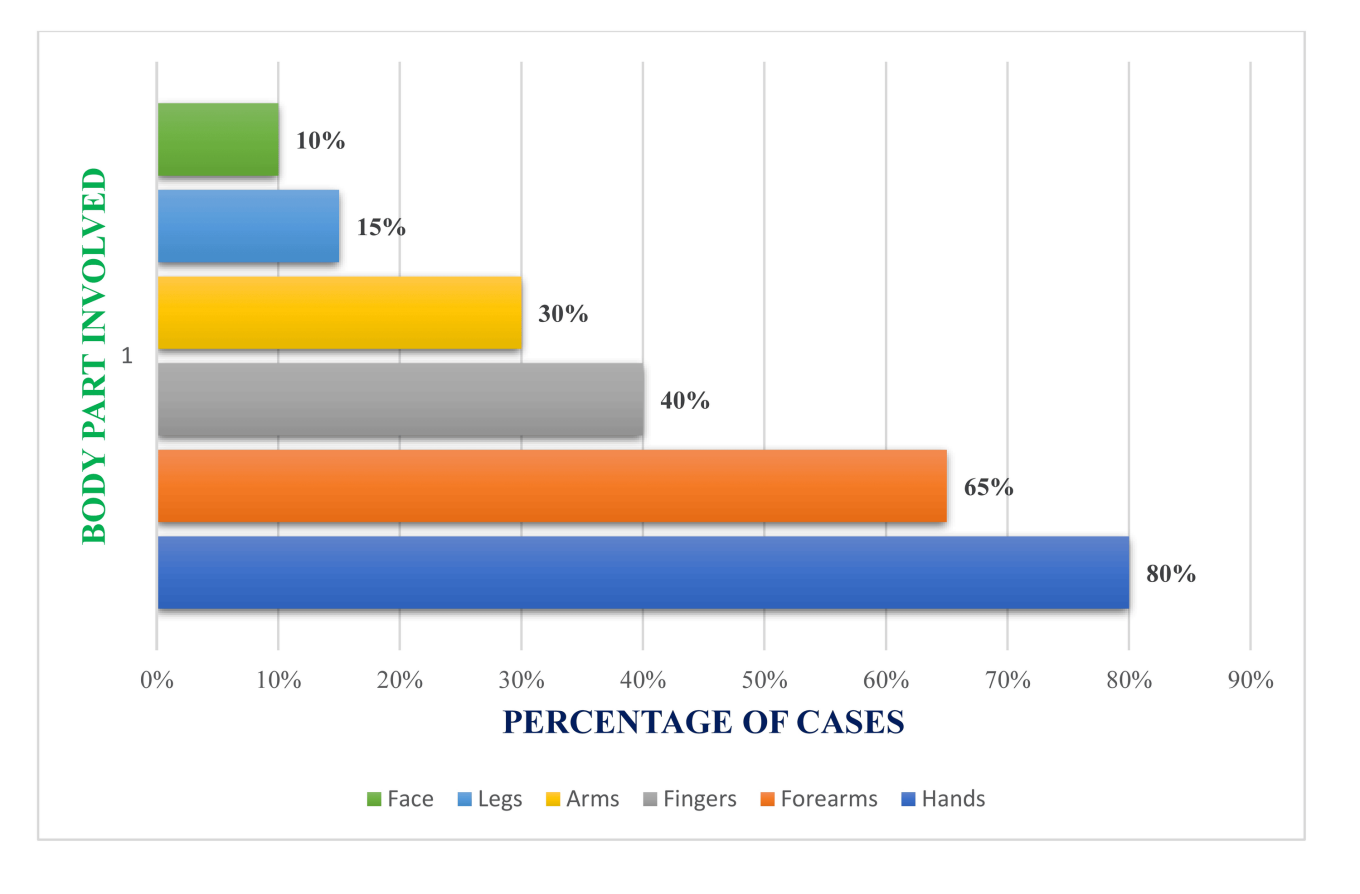
Anatomical distribution of defense injuries

The data presented in Figure 1 support the notion that defense injuries predominantly occur on the upper limbs in 48 (80%) cases (forearms, hands, and arms), as these are instinctively used for protection. Involvement of the lower limbs and face is relatively lower, accounting for six to nine (10 to 15%) cases, which may indicate that attackers generally target upper body regions or that victims prioritize shielding their heads and torsos.

Sharp-edged weapons (knives and blades) were the most frequently used, resulting in a high incidence of incised wounds (36, 60%). Blunt force trauma contributed significantly to the injuries, with contusions (33, 55%), lacerations (27, 45%), and fractures (21, 35%). Firearm-related injuries were the least common (12, 20%), indicating that gun-related violence occurs but is less prevalent than other forms of assault, as depicted in Table 2.

**Table 2:** Types of defense injuries and associated weapons (n=60)

Type of injury	Common weapon used	Frequency	Percentage	p-value	Chi-square value
Incised wounds	Knife, blade	36	60%	0.01	2.71
Contusions	Blunt objects	33	55%	0.02	1.85
Lacerations	Heavy blunt force	27	45%	0.04	0.16
Fractures	Metal rods, clubs	21	35%	0.03	1.19
Gunshot injury	Firearms	12	20%	0.06	5.89

**Figure 2:**
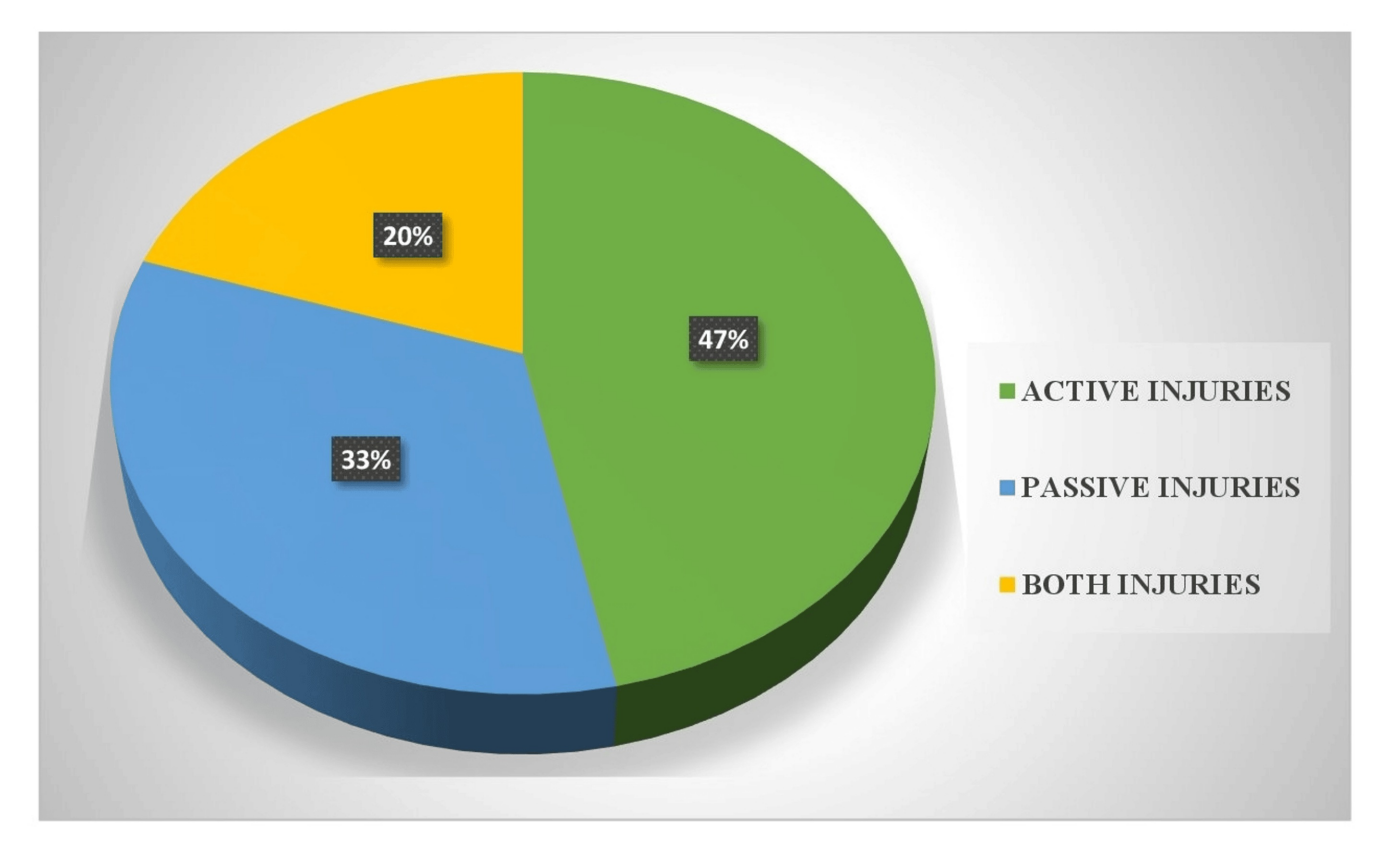
Type of defense wounds (n=60)

Active defense wounds were the most common (28, 47%), indicating that many victims fight back during an attack. Passive defense wounds (20, 33%) are also significant, reflecting defensive gestures such as shielding the body. A combination of both types (12, 20%) suggests variability in defensive responses based on the nature of the attack, as detailed in Figure 2.

## Discussion

The findings of this study align with previous forensic research that suggests defense injuries are most prevalent in cases involving sharp-force trauma. Studies by Chattopadhyay et al. [5] and Shah et al. [6] confirm that incised wounds on the hands and forearms are strong indicators of an attempt to fend off an attacker. Additionally, research by Herman K et al. highlights that parry fractures of the ulna are frequently seen in blunt force trauma in child abuse cases [7].

A study by Kemal et al. found defensive injuries occurred in 31% of homicides and hand wounds were common in homicides in accordance with our analysis. Their work also highlighted that victims with prior self-defense training exhibited a higher incidence of forearm fractures, indicating a more prolonged attempt to resist [8].

Gunshot wounds demonstrated fewer defense injuries in our study, which is consistent with observations by Beardslee et al., who found that firearm-related homicides often involve surprise attacks, leaving victims with little time to react defensively [9]. Similarly, an extensive review by Pardini et al. and Irshad et al. noted that firearm-related defense wounds were often limited to minor abrasions, except in cases of close-range encounters where victims attempted to seize the weapon [10,11].

Our results also correlate with findings by Glass et al., who reported that the absence of defense wounds in strangulation homicides is a strong forensic indicator of restraint before death. This is crucial in differentiating homicide from autoerotic asphyxiation or accidental strangulation [12].

Limitations of the study

This study has several limitations, including a small sample size (60 cases). As a single-center, retrospective study, it relies on existing forensic records, which may contain missing or inconsistent data. The absence of a control group (e.g., suicides and accidents) limits comparative analysis. Additionally, perpetrator characteristics and attack dynamics were not analyzed.

Recommendations

Recommendations include standardized documentation of defense injuries in autopsy reports, forensic medicine training for medical professionals to enhance medico-legal reporting, and further prospective studies incorporating biomechanical analysis of defense wounds.

## Conclusions

This study highlights the forensic significance of defense injuries in homicide cases. The presence, type, and distribution of these injuries assist in crime reconstruction and legal investigations. Proper documentation and interpretation of such injuries are essential for determining the nature of the assault, the weapon used, and the victim's resistance.
